# Distinct activation mechanisms underlie ligand efficacy at a GPCR

**DOI:** 10.64898/2026.10.12.758633

**Published:** 2026-10-18

**Authors:** Matthew K. Howard, Eve J. Fine, Karthik Srinivasan, Daniel D. Richman, Jerome Freudenberg, Zara Weinberg, Jingyou Rao, Ziyue Zou, Christian Macdonald, Patrick Rockefeller Grimes, Balazs R. Varga, James S. Fraser, Mark Von Zastrow, Susruta Majumdar, Harold Pimentel, Justin English, Ron O. Dror, Aashish Manglik, Willow Coyote-Maestas

## Abstract

Ligands that bind to the same G protein-coupled receptor (GPCR) can produce widely different levels of signaling. These differences in efficacy are central to pharmacology, yet how a receptor sequence determines signaling level remains incompletely understood. Here we develop a deep mutational pharmacology approach to measure how each residue in the µ-opioid receptor (µOR) shapes responses to 15 opioids, yielding ∼400,000 measurements of variant effects on signaling and expression, including full concentration-response series for three agonists. Mutational effects on potency are strongest near the orthosteric pocket, whereas effects on efficacy extend through the transmembrane core and intracellular face. Ligands share many functional determinants, but sensitivity to mutation differs with efficacy. As efficacy decreases, the residues required for signaling shift from the binding pocket towards the intracellular face; partial agonists therefore depend on residues that are dispensable for full agonists. Mutations can broadly increase receptor responses, allowing antagonists such as naloxone to produce signaling comparable to a full agonist and increasing the efficacy of partial agonist ligands. Combined mutations can also reorder the relative efficacies of ligands, showing that sequence changes reshape pharmacological differences rather than simply increasing or decreasing all responses together. These findings support a model in which ligands of different efficacy activate the receptor through distinct allosteric mechanisms which are determined by residues distributed across the receptor. Mapping these contributions across diverse ligands with deep mutational pharmacology provides a way to understand how one receptor converts molecular recognition into a range of signaling outputs.

